# Response to Ponamgi *et al.* Comments on Khalighi *et al.* Takotsubo Cardiomyopathy: A Long Term Follow-up Shows Benefit with Risk Factor Reduction. *J. Cardiovasc. Dev. Dis.*, 2015, *2*, 273–281.

**DOI:** 10.3390/jcdd3010004

**Published:** 2016-01-26

**Authors:** Mohammad Umar Farooq, Koroush Khalighi

**Affiliations:** 1 Department of Cardiovascular Disease, Easton Hospital Easton, PA Clinical Professor of Medicine, Drexel University, School of Medicine Easton Cardiovascular Associates 2001 Fairview Ave, Easton, PA 18042, USA; Mohammad_Farooq@chs.net; 2 Department of Medicine, Easton Hospital, 250 South 21 street, Easton, PA 18042, USA

We appreciate the thorough response given by Ponagmi *et al.* [1], who rightly point out that the pathophysiology and modifiable risk factors of Takotsubo Cardiomyopathy (TC) have yet to be unequivocally established. The data from our study corroborates the statement made that emotional/physical stressors that can trigger a severe catecholamine surge may be the most important risk factor for TC. However, our concern stems from published data demonstrating that the probability of recurrence and of future cardiac events is not necessarily low. Ionesco *et al.* found that 52% of the cohort reached the primary end point of a combination of all-cause death, cardiogenic shock, sudden cardiac death, and re-hospitalization for cardiac reasons [2]. Elesber *et al.* found that the recurrence of TC was 10% in the cohort of 100 patients [3]. While we are limited by sample sizes, such data indicates that TC patients may represent a cohort that is predisposed to cardiogenic insult from catecholamine overdrive, and would benefit from prophylactic and protective therapeutic strategies. Thus, we should be wary of simply assuming a low long-term recurrence rate once patients recover from the initial insult, and carefully manage such patients.

Improved diet, exercise, beta blockers, and angiotensin converting enzyme (ACE) inhibitors may all contribute protective effects to the heart against catecholamine surges and stress. While limited by sample size and potential selection bias, we did see a correlation between improved long-term outcomes and optimal medical therapy [4]. This suggests that the careful management of these patients may improve their long-term outcomes, but because this study is limited by small sample sizes, this needs to be explored further.
